# Supplementary material for: Large magneto-Seebeck effect in magnetic tunnel junctions with half-metallic Heusler electrodes
Source: Nat Commun. 2017 Nov 20;8:1626. doi: 10.1038/s41467-017-01784-x (PMC5696366; doi:10.1038/s41467-017-01784-x)
Supplement: Supplementary file 1 — Supplementary Information [file 41467_2017_1784_MOESM1_ESM.pdf]

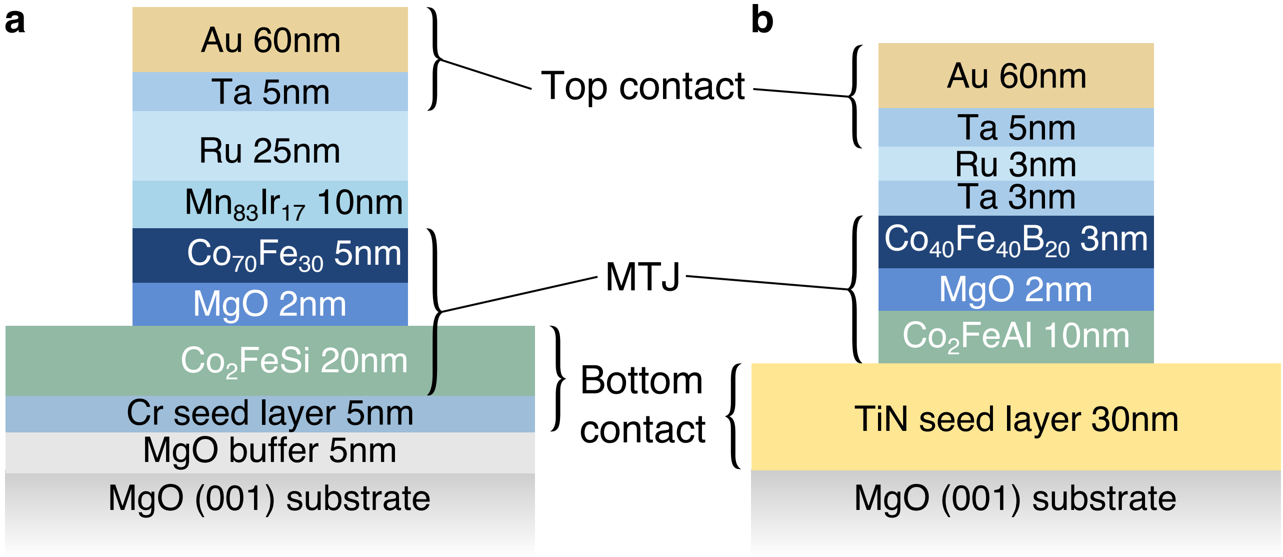

Supplementary Figure 1. **Heusler based MTJs.** **a** Top pinned Co-Fe/MgO/Co_2FeSi MTJs. The Co_2FeSi layer also serves as bottom contact. **b** Co-Fe-B/MgO/Co_2FeSi MTJs. Here, the TiN seed layer provides the bottom contact.

Supplementary Note 1. PREPARATION OF HEUSLER BASED MTJS

The layer stacks for the Co_2FeAl MTJs are produced by magnetron sputtering. The stacks consist of the following layers (thickness in nm) MgO(substrate): / TiN(20) / Co_2FeAl(10) / MgO(2) / $Co_{40}Fe_{40}B_{20}$(5) / Ta(3) / Ru(3). The TiN seed layer is prepared by reactive sputtering in a Bestec GmbH sputter deposition chamber with a base pressure of $5 \cdot 10^{-10}$ mbar. The exact process parameters can be found in Reference[1]. Afterwards, the sample is transferred to a Leybold CLAB sputtering tool (base pressure 10^{-8} mbar) without vacuum break to deposit the remaining layers at room temperature. We apply RF sputtering from a composite target for the MgO barrier and DC sputtering from composite targets for the Co_2FeAl and the $Co_{40}Fe_{40}B_{20}$. The Ta and Ru layers are deposited from elementary targets by DC sputtering. After the deposition we annealed the sample *ex situ* in a vacuum furnace ($p \approx 3 \times 10^{-7}$ mbar) at a temperature of 320°C in a magnetic field of 0.65 T for 1 hour.

The Co_2FeSi based MTJs are produced according to Reference[2]. The used stacks are identical to the stacks used in that earlier work and are prepared with the same equipment. The annealing is the same as for the Co_2FeAl based MTJs.

After deposition and annealing, elliptically shaped MTJs are patterned by e-beam lithography and subsequent ion-milling. For the Co_2FeAl based MTJs we only use the non-ferromagnetic TiN layer as a bottom lead. For the Co_2FeSi based samples also the Co_2FeSi has to be used as bottom lead for providing sufficiently high conductance. Hence, the cross sections of these two sample types look slightly different after etching (Supplementary Figure **1**). For insulation 140 nm of Ta_2O_5 are sputter deposited next to the MTJs, followed by a lift-off procedure. 5 nm of Ta and 60 nm of Au are sputter deposited on top of the MTJs and patterned into contact pads to allow optical access and electrical contact to the MTJs.

Supplementary Note 2. X-RAY INVESTIGATIONS AND TOPOGRAPHY

An extensive study of the quality of the Co_2FeSi films can be found in Reference[2]. These films are produced in the same way as the samples used for the TMS investigations.

For the Co_2FeAl based MTJs, the quality of the buffer layer and the adjacent Co_2FeAl layers is checked by x-ray diffraction (XRD) and atomic force microscopy (AFM) on half MTJ layer stacks prepared in the same way as the complete MTJ stacks (Supplementary Figure **2**). The x-ray investigations are performed in a Phillips X'Pert Pro MPD x-ray diffractometer with Cu K_α radiation at a wavelength of 1.5419 Å. The intensity of the diffracted beam is determined with respect to the incident angle 2θ. A full XRD scan covers the interval of $2\theta = 20° - 125°$. The micrographs are obtained in a Bruker Multimode 5 AFM in contact mode.

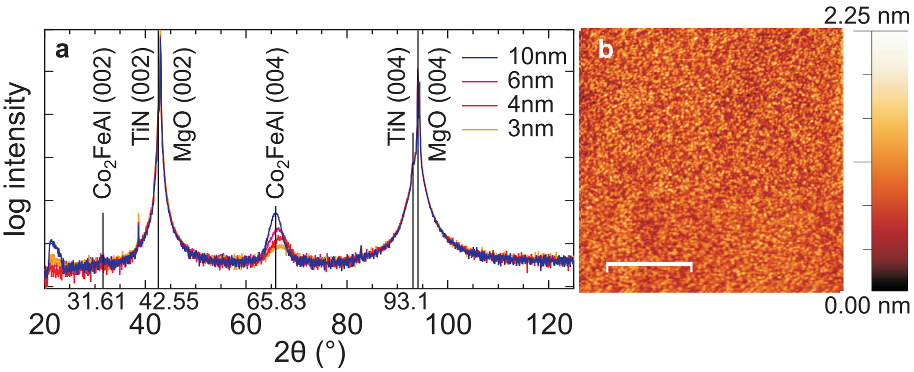

Supplementary Figure 2. Quality of the TiN/Co_2FeAl/MgO layers. a X-ray diffraction data obtained from stacks with different Co_2FeAl thicknesses. The expected diffraction maxima are labeled. **b** Micrograph from an AFM analysis of the layer stack with 10 nm Co_2FeAl with a root-mean-square roughness of 0.25 nm. The scale bar corresponds to a lateral distance of 1.0 μm.

The XRD scans of TiN 30 nm/Co_2FeAl/MgO 2 nm with different Co_2FeAl thicknesses from 3 nm to 10 nm (Supplementary Figure **2**a) unveil that the TiN nearly has the same lattice constant as the MgO substrate, since it is only visible as a small shoulder in the MgO peak. The derived lattice constant for the TiN is (4.25 ± 0.10) Å, which is in good agreement with the literature value of 4.24 Å (see Reference[3]). The XRD results also suggest that a 10 nm Co_2FeAl layer is the optimum choice for the MTJ stacks, as for this thickness the Co_2FeAl (004) peak perfectly matches the expected value of 65.83°. For the Co_2FeAl, we could find a B2 ordering by detecting the (222) and (444) peaks without finding a (111) peak, as it would be present for the $L2_1$ ordered Co_2FeAl.

In addition, the composition of the Heusler electrodes is verified by x-ray fluorescence spectroscopy. The used parameters for the co-sputtering of Co_2FeSi result in a film composition of $Co_{50.5}Fe_{24.5}Si_{25}$. These values are subject to an uncertainty of $\approx 1\%$. Since our setup uses an Al filter, we are not able to check the actual composition of the Co_2FeAl. However, a composite Co_2FeAl target is used and, thus, the final film composition is expected to be close to the target composition.

Furthermore, the micrograph obtained by the AFM investigations (Supplementary Figure **2**b) reveals a low roughness of 0.25 nm for the TiN 30 nm/Co_2FeAl 10 nm/MgO 2 nm layers. This low roughness value is crucial for MTJs with a tunnel barrier of high quality that avoids shortening between the two electrodes through pinholes.

Supplementary Note 3. TEMPORAL EVOLUTION AND SWITCHING BEHAVIOR

A. Co_2FeAl

Supplementary Figure **3**a displays the Seebeck voltage of an elliptically shaped 3 μm $\times$ 1 μm sized Co_2FeAl based MTJ under 150 mW laser power at a laser modulation frequency (on/off) of 13 Hz. Since the spot diameter of the laser on top of the MTJ is $\approx 10\,\mu$m, a homogeneous illumination of the MTJ is ensured. A sharp switching of the Seebeck voltage between $-217\,\mu$Vin the P and $-434\,\mu$V in the AP state of the MTJ is observed. This yields a TMS ratio of -100%. It is noteworthy, that the Seebeck voltage of the Co_2FeAl based MTJs is negative, which is different from the Co-Fe-B MTJs observed by Walter *et al.*[4], Liebing *et al.*[5], and Boehnke *et al.*[6]. Furthermore, the generated Seebeck voltages are much higher, than for the Co-Fe-B based MTJs studied with the same laser setup in References[4,6,7].

The Seebeck coefficients are calculated from the simulated temperature difference across the MgO barrier of 390 mK (cf. Supplementary Note 7 A) and the experimentally determined voltages at a laser power of 150 mW. Seebeck coefficients of $S_\mathrm{P} = 556\,\mu$V/K and $S_\mathrm{AP} = 1113\,\mu$V/K are obtained for the P and the AP state, respectively. These values are comparable to the values in Table I obtained from the 2 μm $\times$ 1 μm MTJ.

The abrupt changes of the Seebeck voltage occur at the same field values and with the same shape as the switching of the resistance of the MTJ. The similarities are nicely seen when the resistance data is plotted on top of the Seebeck voltage (Supplementary Figure **3**a). The resistance, obtained at 10 mV bias voltage, changes between 5.45 MΩ in the P state and 10.87 MΩ in the AP state, yielding a TMR ratio of 99%.

The similar switching behaviors of the resistance and the Seebeck voltage are an evidences that the reversal of the magnetic orientation of the ferromagnetic electrodes causes the change of the Seebeck voltage. Of course, the

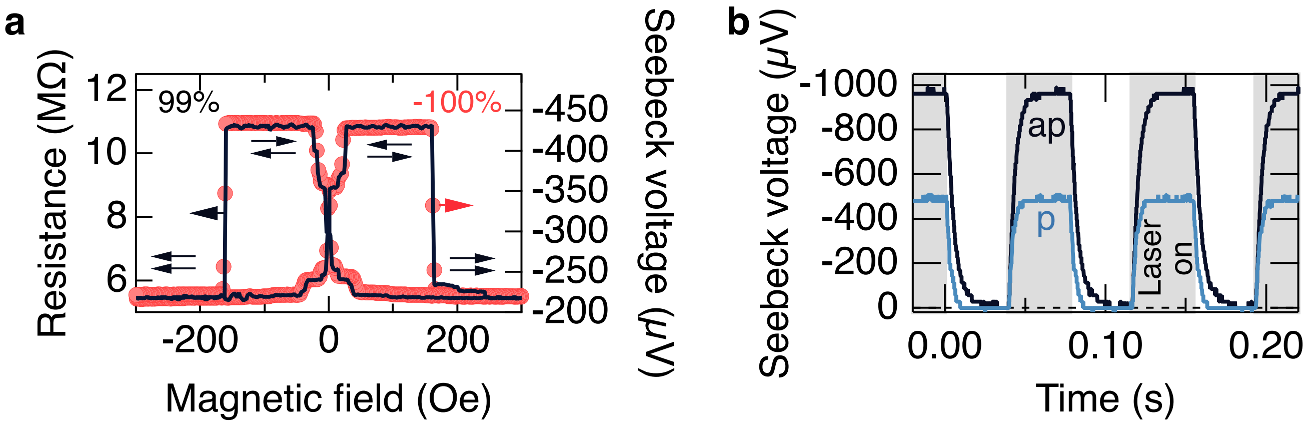

Supplementary Figure 3. **Seebeck voltage of a Co_2FeAl based MTJ. a** The resistance (left axis) at 10 mV bias voltage and the Seebeck voltage (right axis) at 150 mW laser power switch at the same fields, i.e., when the magnetic orientation of the ferromagnetic layers changes between P and AP. **b** Temporal evolution of the Seebeck voltage between laser on/off in the P and AP state.

fundamental origin is the change of the transport coefficients of the MTJ under magnetization reversal (cf. Reference[8]).

Since a modulated heating source in combination with high-resistive MTJs is used, it is crucial to monitor the temporal evolution of the signal. For the correct detection of the Seebeck voltage by the lock-in amplifier, it is highly important that the Seebeck signal saturates after the laser has been switched on or off. During laser on the voltage has to reach a constant plateau, whereas during laser off it should drop back to zero. The rise and fall times of the signal are dependent on the resistance and capacitance of the MTJ[6]; the higher the resistance, the slower is the saturation. Supplementary Figure **3**b displays oscilloscope traces of the Seebeck signal that reveal a saturation of the voltage in the P state after 10 ms and in the AP state after 15 ms when the laser is switched on or off. The increased saturation time in the AP state is attributed to the increased resistance. Accordingly, the modulation of the laser heating with 13 Hz, corresponding to a laser on period of ≈ 38 ms, is slow enough to allow a correct lock-in detection. The higher values of the voltages detected by the scope compared to the lock-in amplifier are due to the difference in the detection techniques. The scope displays the peak-to-peak voltages, whereas the lock-in amplifier displays the root-mean-square value of the first harmonic of the signal[6]. For a conversion to the lock-in-signal, the oscilloscope signal has to be multiplied by a factor of 0.45. As the oscilloscope traces are recorded in the P and AP state of the MTJ, the temporal traces also disclose the difference of the Seebeck voltage between the magnetic states of the MTJ. The TMS ratio obtained from this difference amounts to $\approx -100\%$. Hence, it is of the same size as the TMS ratio obtained from the lock-in measurements in Supplementary Figure **3**a. This good agreement is another proof for the reliability of the correct voltage detection by the lock-in technique.

B. Co_2FeSi

The Co_2FeSi MTJs are equipped with an antiferromagnetically pinned top Co-Fe electrode. The pinning prevents the Co-Fe electrode from switching at low external magnetic fields. The higher fields needed to reverse the magnetization of the pinned layer exceed the field provided by the electromagnet in the optical setup[6]. Hence, it is only possible to record minor loops of the MTJs, i.e., reversing the magnetization of the unpinned Co_2FeSi bottom electrode while keeping the magnetization of the Co-Fe electrode constant, and receiving a signal that resembles the hysteresis loop of this layer.

The TMR and TMS loops of an elliptical $2\,\mu$m $\times$ $1\,\mu$m Co_2FeSi based MTJ with a 2 nm MgO tunnel barrier and a $Co_{70}Fe_{30}$ counter electrode are displayed in Supplementary Figure **4**a. The switching of the resistance between 4.00 MΩ in the AP state and 2.15 MΩ in the P state is clearly visible. The resulting TMR ratio amounts to 86%. The Seebeck voltage obtained at the same MTJ with 150 mW laser power exhibits a similar switching behavior as the resistance. It changes at the same fields between $-664\,\mu$V in the AP state and $-370\,\mu$V in the P state. This change yields a TMS ratio of -80%. Hence, the ratio is only slightly smaller than the values obtained for the Co_2FeAl based MTJs.

Supplementary Figure **4**b features the temporal evolution of the Seebeck signal under 150 mW laser radiation with

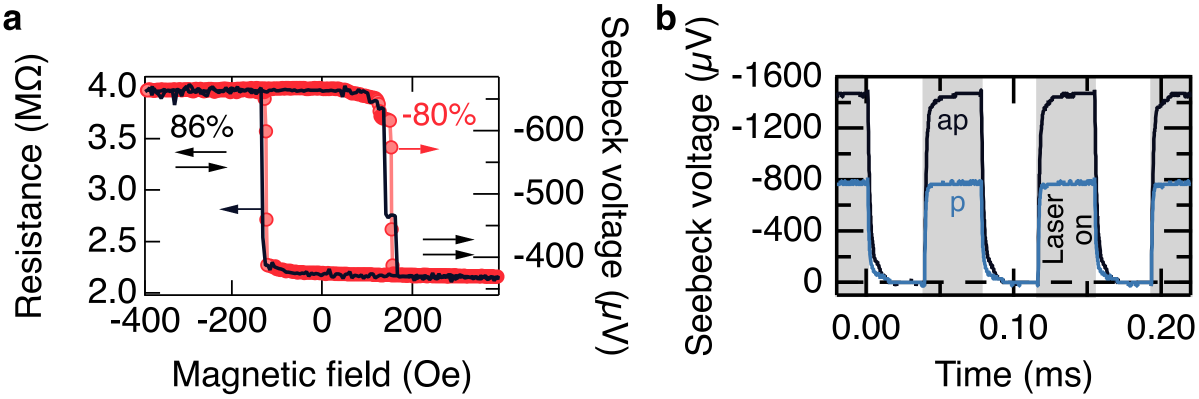

Supplementary Figure 4. **Seeebeck voltage of a Co_2FeSi based MTJ. a** The resistance (left axis) at 10 mV bias voltage and the Seebeck voltage (right axis) at 150 mW laser irradiation switch at the same magnetic fields, i.e., when the magnetization of the Co_2FeSi layer is reversed. **b** Temporal evolution of the Seebeck voltage at 150 mW laser power between laser on/off at a modulation frequency of 13 Hz.

an on/off modulation at a frequency of 13 Hz. The obtained TMS ratio is -92%, which is slightly higher than the value obtained by the lock-in measurement. The peak-to-peak voltages of $\hat{V}_P = -1471\,\mu$V and $\hat{V}_{AP} = -763\,\mu$V correspond to root-mean-square voltages of the first harmonic detected by the lock-in-amplifier of $V_P = -661\,\mu$V and $V_{AP} = -343\,\mu$V. These values are in a similar range as the experimentally obtained lock-in data. Furthermore, the temporal evolution of the Seebeck voltage obtained at the Co_2FeSi based MTJs reveals that the modulation frequency of 13 Hz is slow enough for the Seebeck voltage to reach saturation after the laser is switched on or off. In the P state the increase and decrease of the signal is faster ($\tau^{P}_{1/2} \approx 0.6$ ms) than in the AP state ($\tau^{AP}_{1/2} \approx 1.1$ ms) of the MTJ, due to the decreased resistance of the MTJ in the P state. These values correspond to the rise times of the signal from 0% to 50% when the heating is switched on. The 0% to 90% rise times are $\tau^{P}_{90\%} \approx 1.6$ ms and $\tau^{AP}_{90\%} \approx 3.6$ ms.

A similar switching of the Seebeck voltage under varying magnetic field is found for a second elliptical MTJ with a size of $3\,\mu m \times 1\,\mu m$. For this MTJ (MTJ b) the TMS ratio reaches an average of -95%, which is higher than for the previously described MTJ (MTJ a), where the ratio only yields an average of -83% (Supplementary Table **1**). A difference is also found in the TMR ratios of the two MTJs. MTJ b has a TMR ratio of 102% whereas MTJ a only produces a TMR ratio of 86%. This observation suggests, that the difference of the TMS ratios is purely based on the less pronounced change of the transport coefficients, i.e., the conductance and the Seebeck coefficient, in MTJ a. Such a behavior is usually evoked by a local variation of the quality of the tunnel barrier. This assumption is supported by the fact that the resistance-area product is decreased for the MTJ with the lower TMR and TMS ratios, indicating the poorer quality of the MgO barrier in MTJ a. A comparison of the most important values is presented in Supplementary Table **1**.

Supplementary Table 1. **Overview of Co_2FeSi based MTJs.** The MTJ with the higher resistance-area product exhibits higher TMR and TMS ratios. The resistance is determined at a bias voltage of 10 mV. The Seebeck voltages are recorded with 150 mW laser power and a spot diameter of $\approx 10\,\mu$m. The TMS ratios are averaged over Seebeck measurements with laser powers between 10 mW to 150 mW.

**MTJ**	size ($\mu m \times \mu m$)	$R_P A$ ($M\Omega\,\mu m^2$)	$R_{AP} A$ ($M\Omega\,\mu m^2$)	**TMR**	V_P (μV)	V_{AP} (μV)	**TMS**
a	2×1	3.36	6.28	86%	−370	−664	-83%
b	3×1	3.93	7.94	102%	−263	−503	-95%

The Seebeck coefficients for MTJ a (cf. Supplementary Figure **4** and Supplementary Table **1**) are $S_P = 948\mu V\,K^{-}1$ and $S_{AP} = 1703\mu V\,K^{-}1$. For MTJ b the values still reach up to $S_P = 674\mu V\,K^{-}1$ and $S_{AP} = 1290\mu V\,K^{-}1$. These values are even higher than for the Co_2FeAl based MTJs.

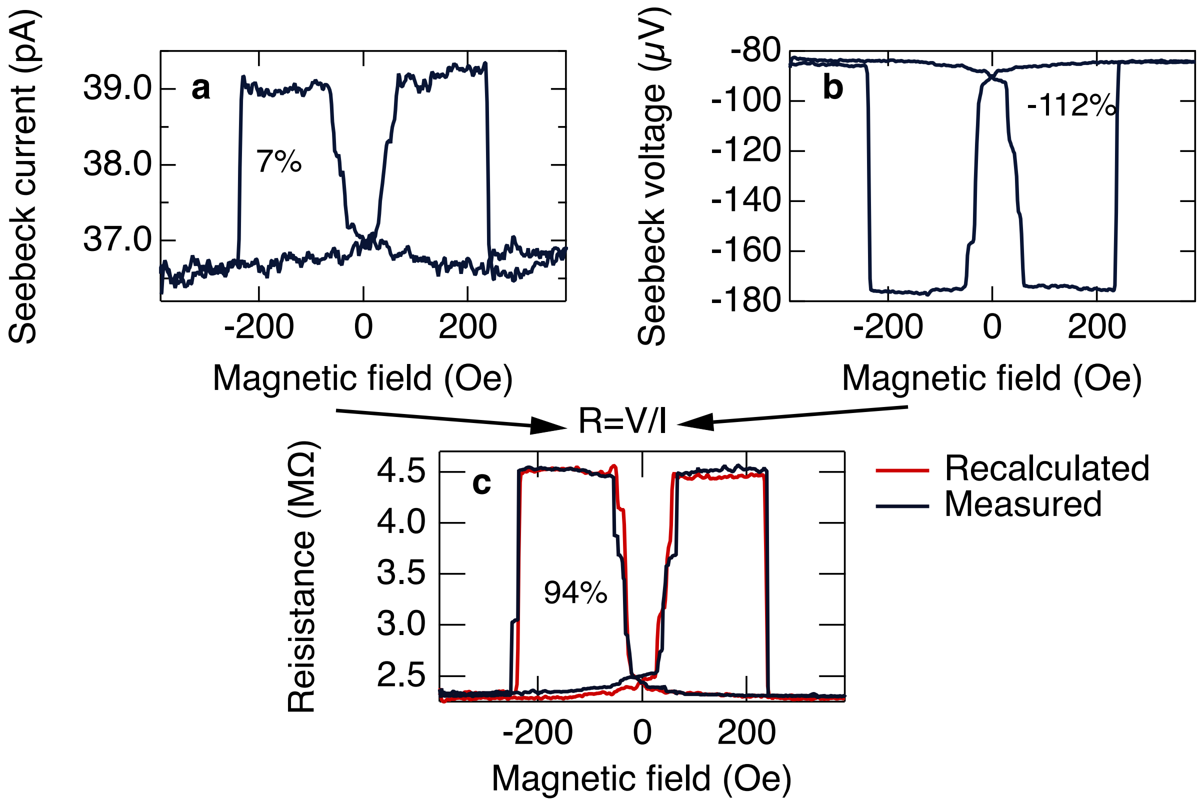

Supplementary Figure 5. **Seebeck current of Co_2FeAl based MTJ. a** Seebeck current recorded at 150 mW laser power. **b** Seebeck voltage of the same MTJ under unchanged irradiation conditions. **c** Directly measured resistance at 10 mV bias voltage and recalculated resistance from Seebeck data in a and b.

Supplementary Note 4. THE SEEBECK CURRENT

Besides the Seebeck voltage, also the Seebeck current of the MTJ is determined. To perform the measurement the heating conditions are not changed, but the lock-in amplifier is directly connected to the MTJ and set to current mode. Derived from Reference[8], the current in such a measurement is expressed by

$$I_{\mathrm{P,AP}} = \frac{1}{R_{\mathrm{P,AP}}} S_{\mathrm{P,AP}} \Delta T = \frac{1}{R_{\mathrm{P,AP}}} (-V_{\mathrm{P,AP}}^{\mathrm{Seebeck}}) \tag{1}$$

$$\Leftrightarrow R_{\mathrm{P,AP}} = -\frac{\left(V_{\mathrm{P,AP}}^{\mathrm{Seebeck}}\right)}{I_{\mathrm{P,AP}}}. \tag{2}$$

This equations reveal that the Seebeck current depends on the change of the Seebeck coefficients $S_{\mathrm{P,AP}}$ and the resistance $R_{\mathrm{P,AP}}$ between the P and AP state of the MTJ. After inserting the Seebeck voltage $V = -S\Delta T$ it resembles Ohm's law (Supplementary Equation 2). Thus, the resistance of the MTJ can be recalculated from the independently recorded Seebeck voltage and Seebeck current data.

A. Co_2FeAl

Supplementary Figure **5**a depicts the Seebeck current obtained from an elliptical Co_2FeAl based MTJ with diameters of $3\,\mu$m $\times$ $1\,\mu$m at 100 mW laser power. The current is switching between 36.6 pA in the P and 39.3 pA in the AP state, resulting in an effect ratio of approximately 7%. The Seebeck voltage determined at the same MTJ without altering the laser irradiation is displayed in Supplementary Figure **5**b. It changes between $-83\,\mu$V in the P state and $-176\,\mu$V in the AP state of the MTJ, yielding a TMS ratio of -112%. Recalculating the resistance from the Seebeck

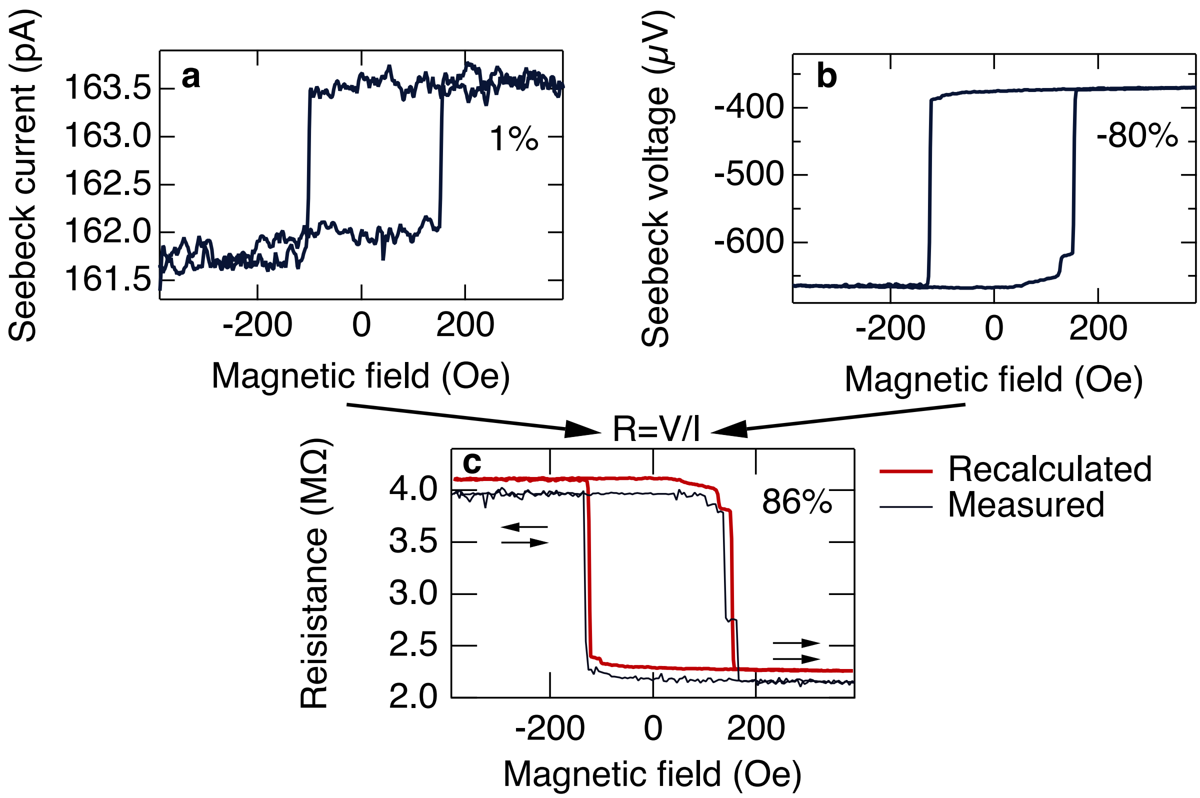

Supplementary Figure 6. **Seebeck current of Co_2FeSi base MTJ. a** Seebeck current recorded at 150 mW. **b** Seebeck voltage of the same MTJ under unchanged irradiation conditions. **c** Directly measured resistance at 10 mV bias voltage and recalculated resistance from Seebeck data in a and b.

current and the Seebeck voltage data in Supplementary Figure **5**a and b, results in a curve that almost perfectly fits the measured resistance displayed in Supplementary Figure **5**c.

A similar agreement of the recalculated and measured resistance is also achieved for Seebeck measurements with 150 mW laser power and on a second similarly shaped MTJ with resistances $R_\mathrm{P} = 5.4\,\mathrm{M\Omega}$ and $R_\mathrm{AP} = 10.8\,\mathrm{M\Omega}$. This MTJ reveals a TMR ratio of 100% and a TMS ratio of -97%.

B. Co_2FeSi

Similar to the experiments performed with Co_2FeAl based MTJs, the Seebeck current of the MTJ is determined and the resistance is recalculated by dividing the Seebeck current by the Seebeck voltage. These values are obtained from independent measurements at the same MTJ under unchanged irradiation conditions.

Supplementary Figure **6**a displays the switching of the Seebeck current of MTJ a in Supplementary Table **1** (elliptical shape of $2\,\mu\mathrm{m} \times 1\,\mu\mathrm{m}$). The current resembles the same switching behavior as the resistance and the Seebeck voltage (Supplementary Figure **6**b). However, the effect ratio only yields 1%. This is attributed to the similar sizes of the TMR and TMS ratios. According to $I = S/R \cdot \Delta T$, the switching of the Seebeck coefficient S and the resistance R cancel out when the two effects are of similar magnitudes.

Supplementary Figure **6**c displays the resistance of the MTJ under varying magnetic field. As before, the difference between the P and AP state of the MTJ is clearly recognizable. Furthermore, the curve of the recalculated resistance from the Seebeck data lies on top of the directly measured resistance, except of a small offset.

As the resistance is correctly recalculated from the Seebeck voltage and current, we draw the conclusion that the Seebeck voltage and current detection of the MTJ are very reliable and reproducible. Independent measurements lead to similar results.

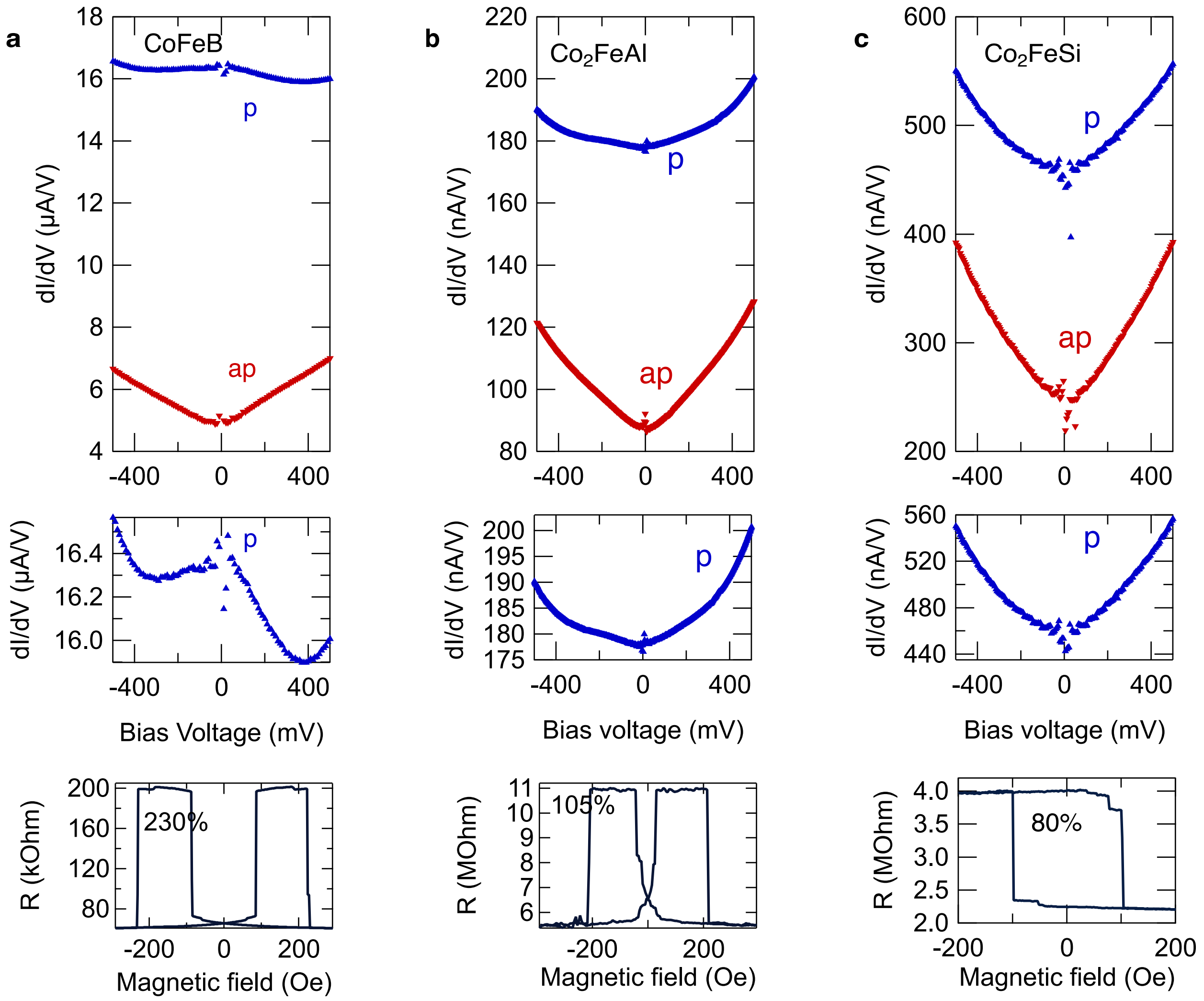

Supplementary Figure 7. **Differential conductance.** Differential conductance (top) and corresponding TMR effect (bottom) of MTJs containing **a** Co-Fe-B, **b** Co_2FeAl and **c** Co_2FeSi.

Supplementary Note 5. DIFFERENTIAL CONDUCTANCE

The differential conductance dI/dV in dependence of the applied bias voltage V reveals information on the energy landscape that the electrons experience when traveling between the two electrodes of a tunnel junction. Particularly the DOSs left and right of the tunnel barrier around the Fermi energy have a significant influence on the shape of the dI/dV vs. V curves. Brinkman has shown that for tunneling between two constant (flat) DOSs through a trapezoidal barrier, the dI/dV curve always exhibits a parabolic shape[9]. This description is no longer valid if the DOS possesses a gap. If the Fermi energy is scanned across a gap in the DOS by changing the bias voltage, the tunneling current will not significantly change although the voltage is increased. This is due to the lack of states in the gap. Only if the energy exceeds the gap energy and states are available, the current will increase again. In the dI/dV curve this can be recognized by a flat section around zero bias voltage leading to a deviation from the predicted parabolic shape.

For all MTJs investigated in this work, a gap or at least a pseudo gap can be found. In the Co-Fe-B/MgO/Co-Fe-B MTJs the gap is introduced by the coherent tunneling through the MgO barrier. In the Heusler compound based MTJs additionally a gap in the Heusler minority DOS is expected. Hence, a deviation from a perfect parabolic shape can be found in all dI/dV curves (Supplementary Figure **7**). For a detailed explanation of the measurements at Co_2FeAl and Co-Fe-B and a comparison of the dI/dV curves to earlier measurements, please refer to Mann *et al.*[10]. For the Co_2FeSi MTJs the deviation from the parabolic shape is hardly visible. This might be due to several facts:

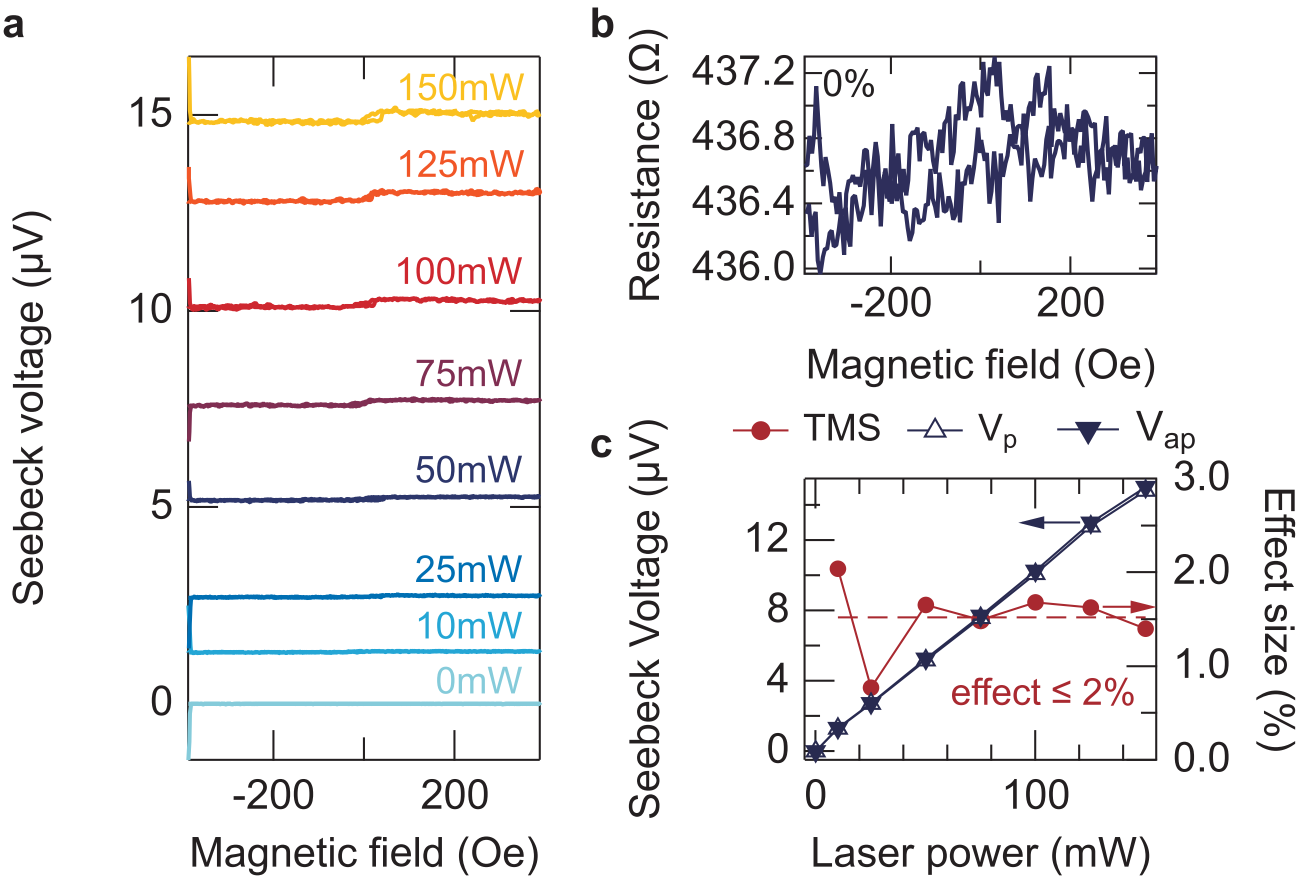

Supplementary Figure 8. **Co_2FeAl based MTJs after dielectric breakdown.** **a** Dependence of the Seebeck voltage for different laser powers, and **b** the resistance on the magnetic field. **c** Dependence of the Seebeck voltage in the P and AP state (left axis), and the effect ratio (right axis) on the laser power.

First, the IV-characteristics was obtained at room temperature. Second, the gap is not a real gap but a pseudo gap. Both circumstances lead to a less pronounced influence of the half-metallic DOS on the dI/dV characteristic. Third, the pseudo gap in the DOS is symmetric with respect to the Fermi energy, which leads to a symmetric dI/dV curve.

However, for a large TMS effect not only the presence of a gap in the DOS is of importance, also its position and the difference between the DOSs that contribute to the thermoelectric transport in the p and ap state have a significant impact. Since the tunneling current is represented by an integration of the DOS or the related transmission T(E) over energy E (cf. Walter *et al.*[4]), the dI/dV curve cannot reveal the important features in the DOS that are necessary to gain a high TMS effect. Nevertheless, all curves show that the asymmetry of the transmission changes when the MTJ is switched from p to ap. This is a necessary ingredient to generate a high TMS effect.

Supplementary Note 6. CONTRIBUTIONS FROM THE LEADS: MTJS AFTER DIELECTRIC BREAKDOWN

To ensure that the Seebeck voltage is indeed generated by the temperature gradient across the MTJ and not, for example, in the leads, the junction is forced into a dielectric breakdown and the remaining Seebeck voltage is determined under unchanged irradiation conditions (Supplementary Figure **8**). To break the 2 nm tunnel barrier, a bias voltage of 4 V is applied to the MTJ. The broken tunnel junction disables the spin-dependent tunneling across the barrier. Hence, after the breakdown of the barrier the TMR effect vanishes in both sample types, the Co_2FeSi and the Co_2FeAl based MTJs (Supplementary Figures **9** & **8**). Simultaneously, the resistance drops from a few MΩ to a few hundred Ω, clearly indicating that the barrier has been destroyed by the voltage stress.

A similar behavior is found for the Seebeck voltage that exhibits almost no response to the external magnetic field. Only for higher laser powers, i.e., higher Seebeck voltages, a hysteresis loop is distinguished from the noise. The origin of the remaining switching might be due to magneto transport phenomena in the ferromagnetic Co_2FeSi bottom lead

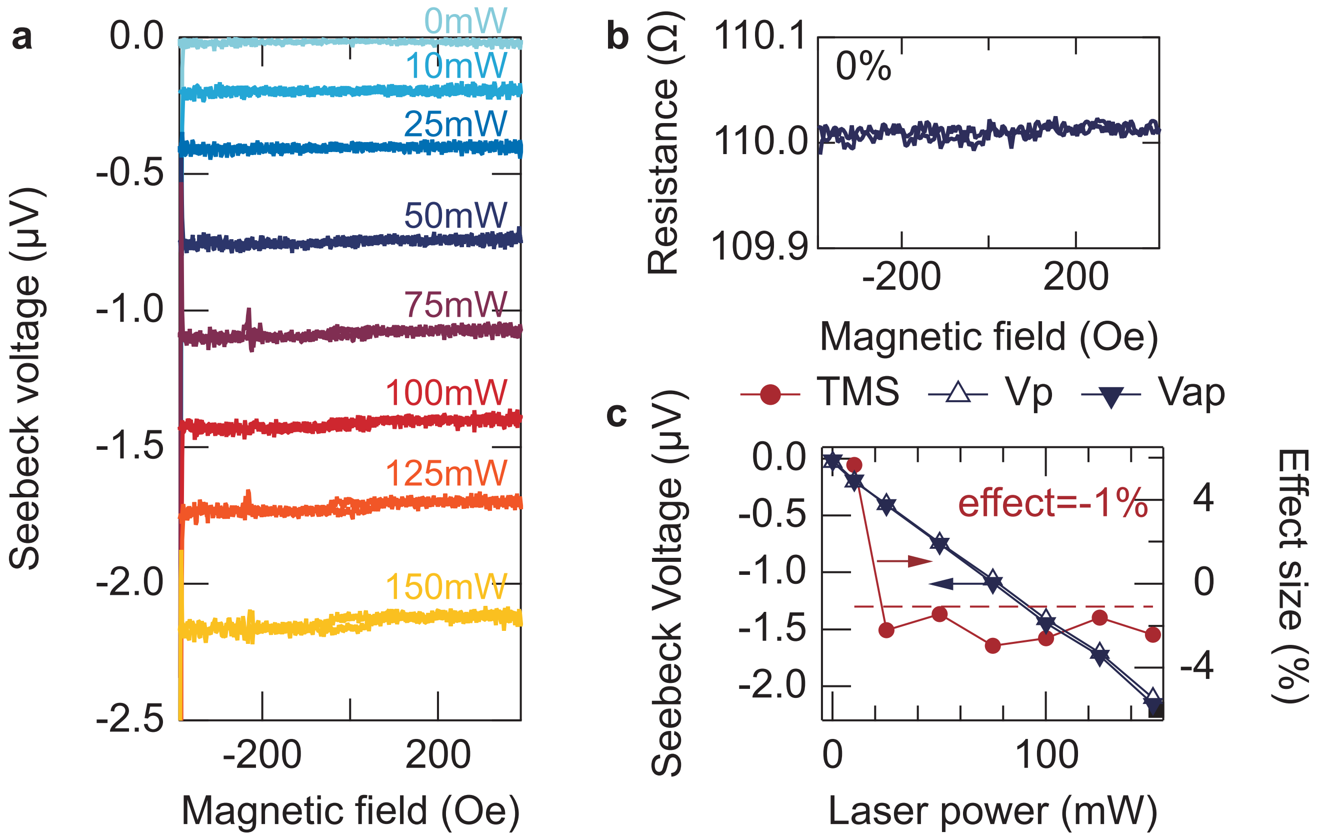

Supplementary Figure 9. Co_2FeSi based MTJs after dielectric breakdown. **a** Dependence of the Seebeck voltage for different laser powers, and **b** the resistance on the magnetic field. **c** Dependence of the Seebeck voltage in the P and AP state (left axis), and the effect ratio (right axis) on the laser power.

or the shortened ferromagnetic electrodes in the Co_2FeAl based MTJs. For both MTJ types we emphasize that the shape of the Seebeck voltage versus magnetic field curves after breakdown of the junction are significantly different from the curves recorded at the same intact junctions. Since the MTJs reveal nearly no switching after the tunnel barrier is forced into a dielectric breakdown, it is guaranteed that the leads do not significantly contribute to the high TMS ratios obtained from the intact MTJs.

Moreover, the remaining Seebeck voltages of the broken MTJs are strongly reduced from several $-100\,\mu$V to a few μV. For the Co_2FeAl based MTJs that possess a TiN bottom lead free from ferromagnetic Heusler material, even the sign of the voltage is reversed. These remaining Seebeck voltages are probably generated by in-plane temperature differences in the bottom lead and not in the MTJs themselves. From this comparison of intact and broken MTJs, we conclude that the high Seebeck voltages and TMS ratios observed in the intact Heusler based MTJs are only generated due to the temperature difference across the MgO barrier.

Supplementary Note 7. COMSOL SIMULATIONS OF THE TEMPERATURES DURING HEATING

COMSOL Multiphysics finite element simulations are performed to determine the rise of the base temperature and the achieved temperature gradient over the MgO barrier, when the laser is applied to the top of the MTJ. The model we use is similar to the one of References[4,6,7], but the material parameters are adjusted according to Supplementary Table **2**. For the simulations the MTJ sizes, the laser spot size and the laser power are chosen according to the values used in the experiments. Furthermore, the influence of the size of the laser spot on the temperature profile in the MTJs is investigated.

Supplementary Table 2. **Material parameters for COMSOL simulations.** If not specified otherwise, the values are taken from Refs.[4,11–13]. The density of the Heusler compounds and the TiN layers are taken from XRR measurements. The thermal conductivities used in the simulations are printed in bold letters. Experimental thin film values are given if available.

Material	$\rho\,(10^3\,\frac{\text{kg}}{\text{m}^3})$	$c_\text{p}\,(\frac{\text{J}}{\text{kg}\cdot\text{K}})$	$\kappa_\text{bulk}\,/\,\kappa_\text{thin}^\text{exp}\,(\frac{\text{W}}{\text{m}\cdot\text{K}})$
Au	19.32	128	**320.0** / 70[14] – 170[15]
Cr	7.15	449	**94.0**
Ru	12.37	238	**117.0**
Ta	16.65	140	**57.0**
Ta_2O_5	8.27	306	**0.2**
$Ni_{80}Fe_{20}$	8.7	460	**19.0**
Ir-Mn	10.18	69.7	**6.0**
Co-Fe-B	8.22	440	**86.7**
MgO	3.58	935	48.0 / **4.0**[16]
SiO_2	2.20	1052	**1.4**
Si	2.33	700	**150.0**
SiN	3.11	700	**35.9**
Co_2FeAl	6.8	424[17]	**20**[18,19]
Co_2FeSi	7.2	424[17]	**20**[18,19]
TiN	5.45	604[20]	**28.8**[?]

A. Heusler compound MTJs

For the simulations of the temperature evolution upon heating in the Heusler compound based MTJs, a round MTJ of 3 μm in diameter is assumed. The laser spot is modeled as a Gaussian beam with a beam waist of 5 μm. This value equals the experimentally determined beam diameter of 10 μm. For the laser with a power of 150 mW, a power of 120 mW is measured at the position of the sample. Hence, this value is used in the simulations.

Supplementary Figure **10** displays the results of the COMSOL simulations for the Co_2FeAl and Co_2FeSi based MTJs. In Supplementary Figure **10**a it can be seen that a temperature gradient is generated across the barrier of the MTJ, pointing from the bottom Heusler electrode (lower temperature) to the top electrode. The gradient across the insulating barrier is much steeper than the gradient generated in the metallic electrodes. This gradient over the barrier is the driving force for the Seebeck induced tunneling of electrons across the electrodes.

Hence, this gradient is used to calculate the Seebeck coefficients from the experimentally determined Seebeck voltages, which is in accordance with other TMS experiments performed by Walter *et al.*[4] and Liebing *et al.*[5,11]. The obtained gradients amount to a ΔT of 390 mK for the laser set to a power of 150 mW for both Heusler based MTJ types.

However, if the temperature change at all interfaces, i.e., over all layers of the MTJ (Supplementary Figure **10**b), is considered, a second much larger gradient is observed in the Co_2FeSi based MTJs. This gradient is attributed to the Mn-Ir pinning layer. Mn-Ir has a much lower heat conductivity than the surrounding layers (cf. Supplementary Table **2**), and hence, supports the generation of a temperature gradient. For the Co_2FeAl based MTJs that do not contain an Mn-Ir layer, no second gradient, that is equally steep as the gradient over the MgO barrier, is observed. The large second gradient in the Co_2FeSi based MTJs makes it necessary to check if the determined voltage indeed is mostly generated by the temperature gradient across the MgO barrier, and not by the second gradient in the samples. This is done by breaking the MgO barrier and determining the remaining Seebeck voltage, as discussed in the main text and in detail in Supplementary Note 6.

For the Co_2FeAl based MTJs simulations with different laser powers are performed (Supplementary Figures **10**c,d). The generated temperature gradients rise linearly with the laser power and range between 30 mK for the laser set to 10 mW and nearly 400 mK for the laser set to 150 mW. Also, the base temperatures for different laser powers rise linearly from room temperature to approximately 350 K at 150 mW laser power. Since the temperature increases linearly with the applied laser power, a linear increase of the Seebeck voltage with laser power is expected. However, due to the simultaneous rise of the base temperature, it is possible to observe the temperature dependence of the Seebeck coefficients. This dependence might lead to a deviation of the Seebeck voltage from the expected ideal linear behavior.

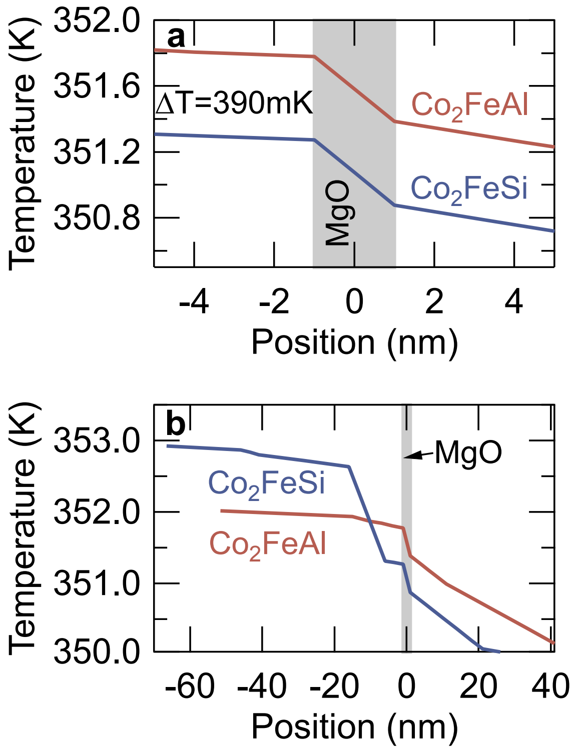

Supplementary Figure 10. **Simulated temperature gradients in the Heusler based MTJs.** **a** ΔT across the 2 nm MgO barrier at 150 mW laser power. **b** Temperature evolution over the whole layer stacks. **c** Dependence of ΔT in the Co_2FeAl based MTJs on the applied laser power. **d** Increase of the base temperature $\overline{T}$ with laser power for the Co_2FeAl based MTJs.

B. Dependence on laser spot diameter

For the Co_2FeAl based MTJs simulations of the temperatures with different sizes of the laser spot have been conducted (Supplementary Figure **11**). The smallest diameter is chosen to be $4\,\mu$m and the largest to be $40\,\mu$m. Within this range, a significant drop of the temperature difference across the MgO barrier from $\Delta T = 800\,$mK for the smallest beam size to 50 mK for the largest beam is obtained. Simultaneously, the base temperature decreases from 390 K to 310 K.

These results reveal how drastically the size of the laser spot influences the Seebeck effect measurements. Hence, the beam size has to be carefully checked before or after each measurement.

SUPPLEMENTARY REFERENCES

[1] Niesen, A. *et al.* Titanium nitride as a seed layer for Heusler compounds. *J. Appl. Phys.* **118**, 243904 (2015).

[2] Sterwerf, C., Meinert, M., Schmalhorst, J.-M. & Reiss, G. High TMR Ratio in Co_2FeSi and Fe_2CoSi Based Magnetic Tunnel Junctions. *IEEE Trans. Magn.* **49**, 4386-4389 (2013).

[3] Kato, A. & Tamari, N. Crystal growth of titanium nitride by chemical vapor deposition. *J. Cryst. Growth* **29**, 55-60 (1975).

[4] Walter, M. *et al.* Seebeck effect in magnetic tunnel junctions. *Nat. Mater.* **10**, 742-746 (2011).

[5] Liebing, N. *et al.* Tunneling Magnetothermopower in Magnetic Tunnel Junction Nanopillars. *Phys. Rev. Lett.* **107**, 177201 (2011).

[6] Boehnke, A. *et al.* Time-resolved measurement of the tunnel magneto-Seebeck effect in a single magnetic tunnel junction. *Rev. Sci. Instrum.* **84**, 063905 (2013).

[7] Boehnke, A. *et al.* On/off switching of bit readout in bias-enhanced tunnel magneto-Seebeck effect. *Sci. Rep.* **5**, 8945 (2015).

[8] Czerner, M., Bachmann, M. & Heiliger, C. Spin caloritronics in magnetic tunnel junctions: Ab initio studies. *Phys. Rev. B* **83**, 132405 (2011).

[9] Brinkman, W. F., Dynes, R. C. & Rowell, J. M. Tunneling Conductance of Asymmetrical Barriers. *J. Appl. Phys.* **41**, 1915-1921 (1970).

[10] Mann, A. *et al.* Insights into Ultrafast Demagnetization in Pseudogap Half-Metals. *Phys. Rev. X* **2**, 041008 (2012).

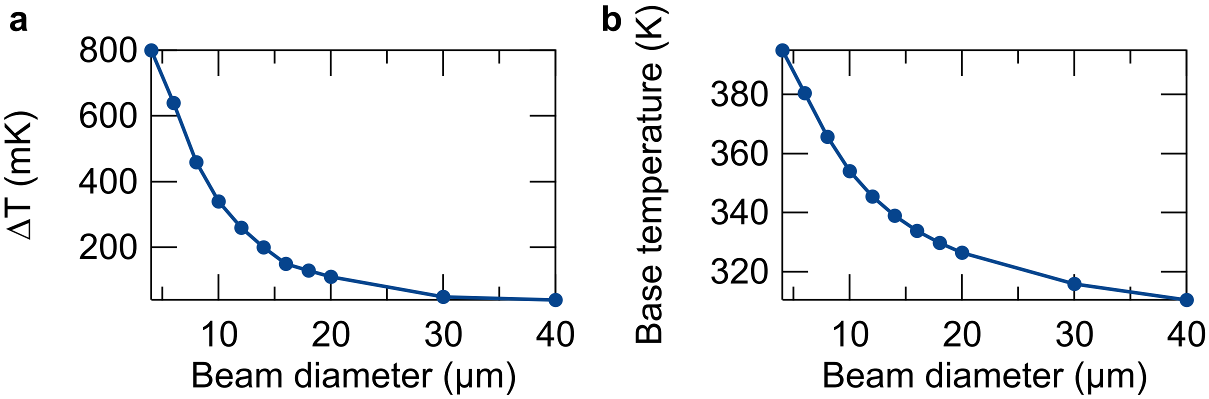

Supplementary Figure 11. Dependence of the temperatures on the beam size in the Co_2FeAl based MTJs. a The temperature difference across the MgO barrier. **b** The base temperature in the center of the MgO barrier. The simulations are performed for the laser set to a power of 150 mW.

[11]Liebing, N. *et al.* Determination of spin-dependent Seebeck coefficients of CoFeB/MgO/CoFeB magnetic tunnel junction nanopillars. *J. Appl. Phys.* **111**, 07C520 (2012).

[12]Papusoi, C., Sousa, R., Herault, J. Prejbeanu, I. L. & Dieny, B. Probing fast heating in magnetic tunnel junction structures with exchange bias. *New J. Phys.* **10**, 103006 (2008).

[13]Beecher, C., Dinwiddie, R. B., Abeel, A. M. & Lowden R. A. The thermal conductivity of silicon nitride with molybdenum disilicide additions (ed Tong, T. W.) (Technomic Publishing Company, 1994).

[14]Zink, B. L., Revaz, B., Cherry, J. J. & Hellman, F. Measurement of thermal conductivity of thin films with a Si-N membrane-based microcalorimeter. *Rev. Sci. Instrum.* **76**, 24901 (2005).

[15]Zhang, Q. G., Cao, B. Y., Zhang, X., Fujii, M. & Takahashi, K. Influence of grain boundary scattering on the electrical and thermal conductivities of polycrystalline gold nanofilms. P*Phys. Rev. B* **74**, 134109 (2006).

[16]Lee, S. M., Cahill, D. G. & Allen, T. H. Thermal conductivity of sputtered oxide films. *Phys. Rev. B* **52**, 253-257 (1995).

[17]Bentouaf, A. & Hassan, F. E. H. Structural, electronic, magnetic and thermodynamic properties of full Heusler compound Co_2VSi: Ab initio study*J. Magn. Magn. Mater.* **381**, 65-69 (2015).

[18]Shiomi, J., Esfarjani, K. & Chen, G. Thermal conductivity of half-Heusler compounds from first-principles calculations. *Phys. Rev. B* **84**, 104302 (2011).

[19]Lue, C. S. & Kuo, Y.-K. Thermoelectric properties of the semimetallic Heusler compounds $Fe_{2x}V_{1+x}M$ (M=Al, Ga). *Phys. Rev. B* **66**, 085121 (2002).

[20]Chase Jr., M. W. *NIST-JANAF Thermochemical Tables, Fourth Edition. J. Phys. Chem. Ref. Data, Monograph* **9**, 1-1951 (1998).
